# Supplementary material for: Chloranthus genome provides insights into the early diversification of angiosperms
Source: Nat Commun. 2021 Nov 26;12:6930. doi: 10.1038/s41467-021-26922-4 (PMC8626473; doi:10.1038/s41467-021-26922-4)
Supplement: Supplementary file 1 — Supplementary Information [file 41467_2021_26922_MOESM1_ESM.pdf]

Chloranthus genome provides insights into the early diversification of angiosperms

Guo *et al.*

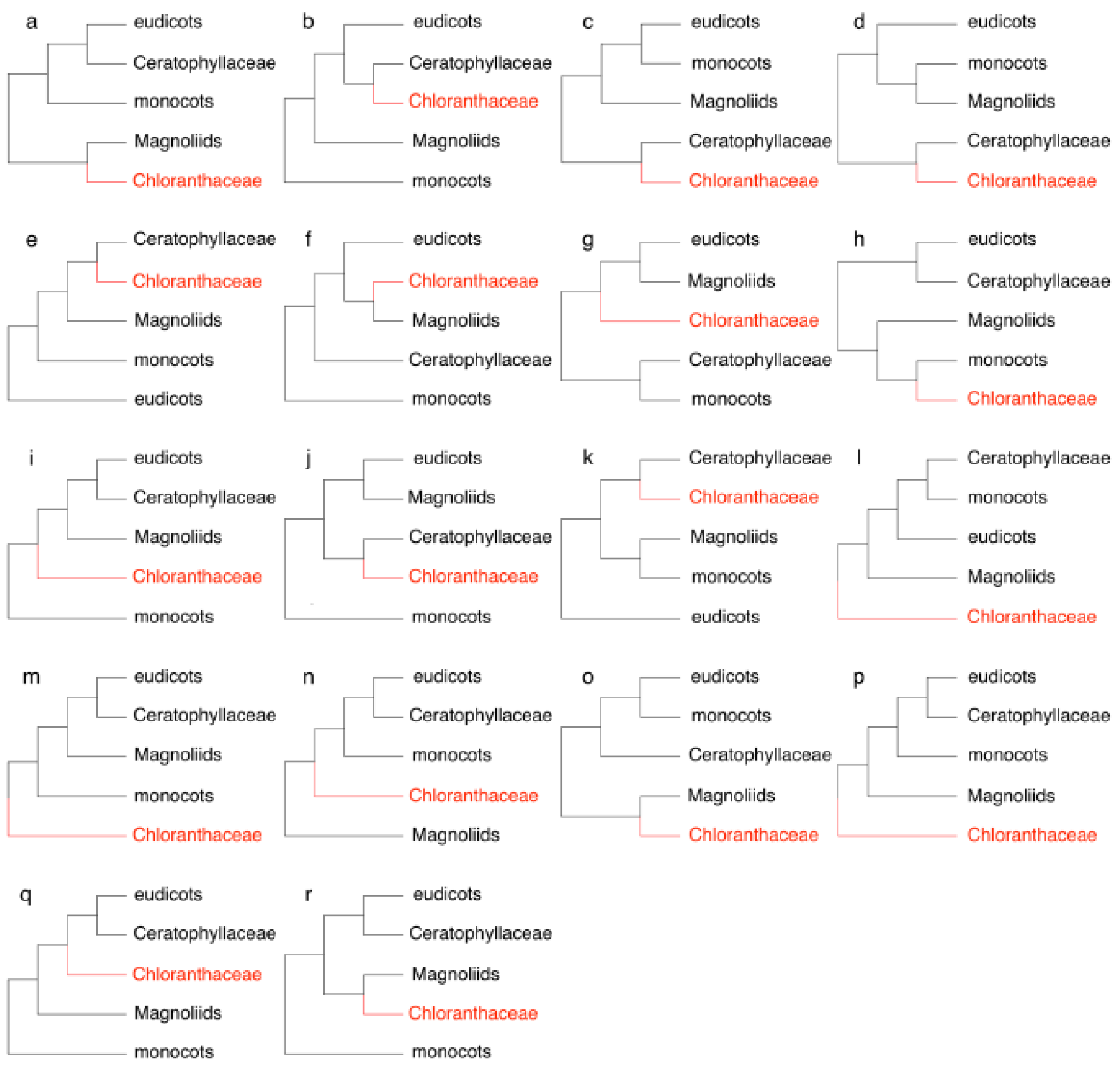

Supplementary Fig. 1. Various alternative phylogenetic hypotheses showing the placement of Chloranthaceae in previous studies.

(a) refs 1-3; (b) ref 4; (c) ref 5; (d) ref 6; (e) ref 7; (f) ref 8; (g) refs 9-11; (h) refs 9,12; (i) ref 9; (j) ref 9; (k) ref 9; (l) refs 3,9; (m) ref 13; (n) ref 2; (o) ref 14; (p) ref 15; (q) ref 16; and (r) ref 17.

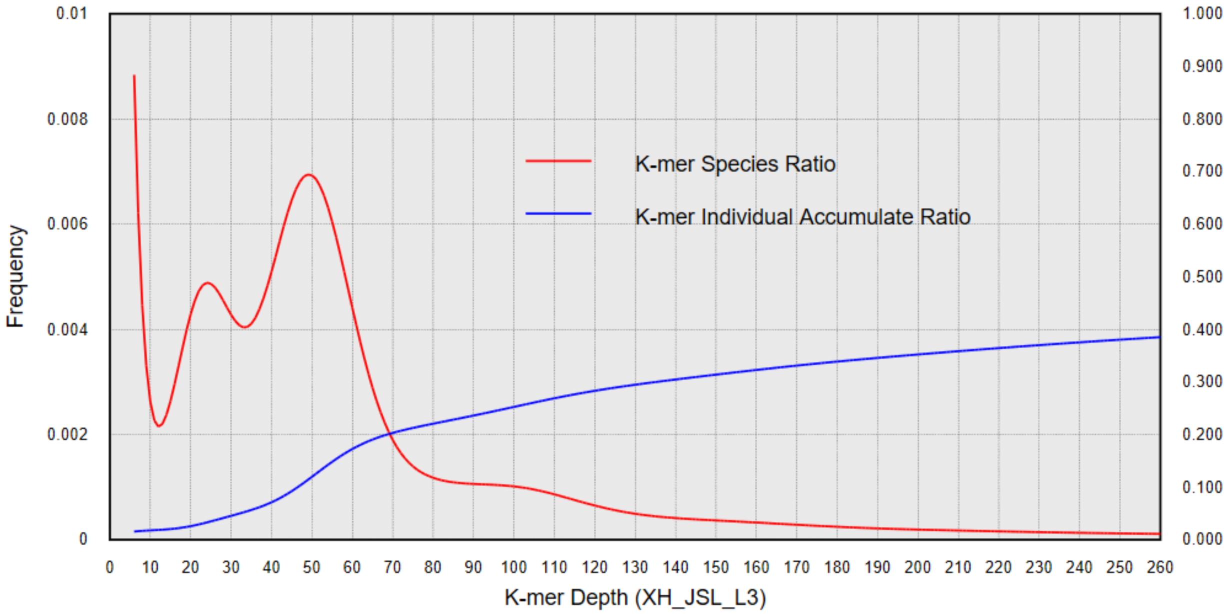

Supplementary Fig. 2. Estimation of the genome size of *C. spicatus* by K-mer analysis. The frequency and sequencing depth of 17 k-mer were plotted. Genome size was estimated using the primary peak depth (Peak Value = 49), and heterozygosity was estimated by the second peak. The red line represents the ratio of k-mer species with difference depths, while the green line represents the accumulated ratio of individual k-mers.

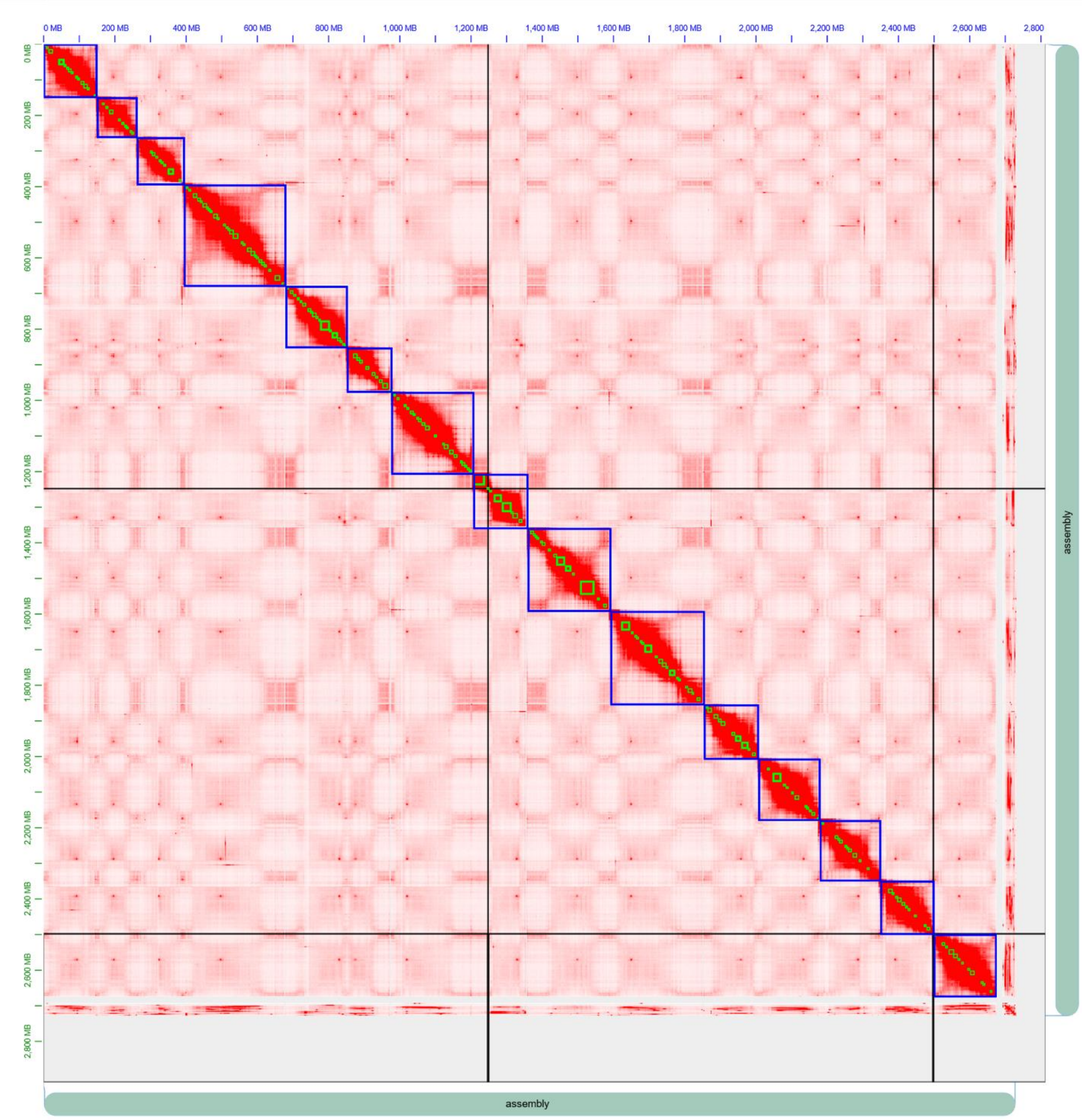

Supplementary Fig. 3. Hi-C map of the *C. spicatus* genome showing genome-wide all-by-all interactions. The map shows a high resolution of individual chromosomes that were scaffolded and assembled independently. Abundant intrachromosomal contacts were also observed. Interchromosomal contacts were also found but with much decreased intensity.

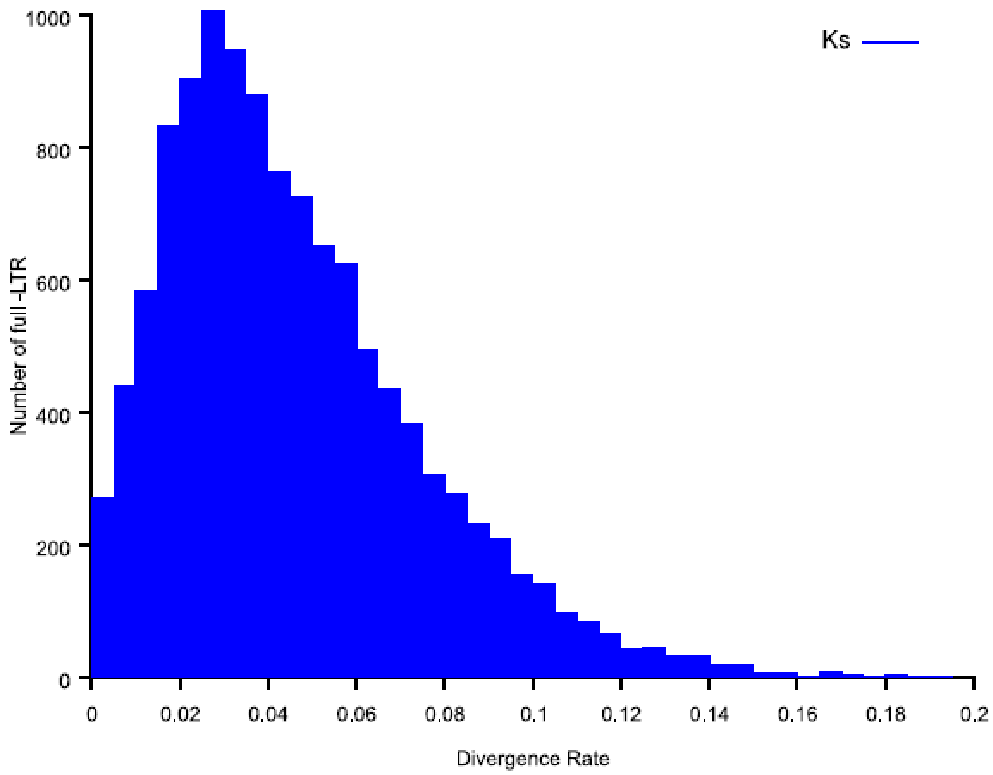

Supplementary Fig. 4. Estimated distribution of full-LTR Insertion Times in the *C. spicatus* genome. *Ks* distributions of the full-LTR in the *C. spicatus* genome was plotted by a window of 0.005, and a *Ks* peak was found at 0.03. We assumed a mutation rate of 1.51 x 10^{-9} bases per year[18], resulting in an insertion time of approximately 9.9 Ma.

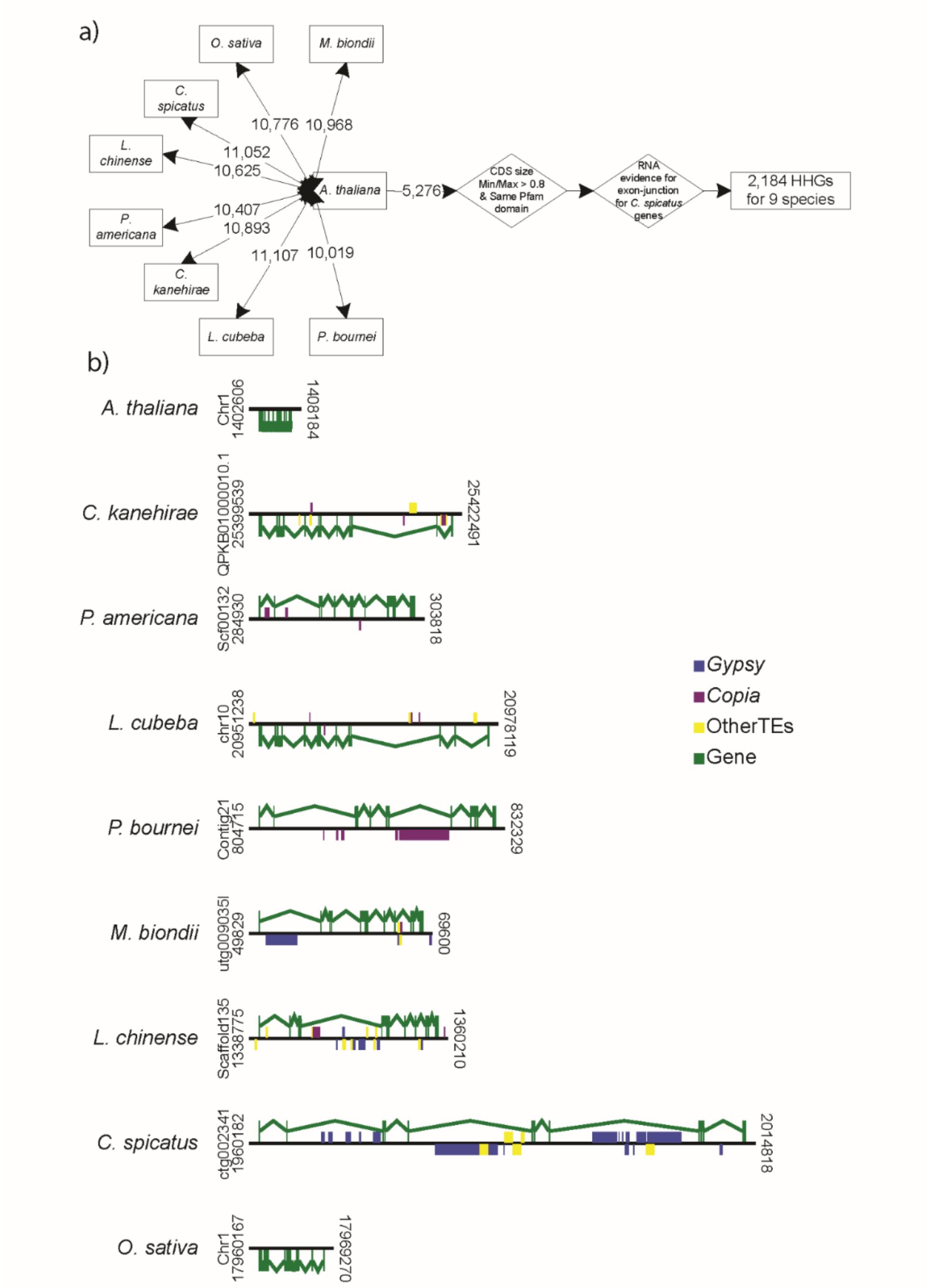

Supplementary Fig. 5. Gene structure and inserts for repetitive sequences. (a) The retrieval of homologous genes from nine species. To compare the component in the intron regions of the same gene families, homologs were retrieved from seven magnoliids and two well-characterized angiosperm genomes *A. thaliana*, and *O. sativa* (Fig S1). We firstly used BLAST to get a set of high scoring pairs (HSPs) with *A. thaliana* as reference, and 5276 genes were consistently present in each species. Then genes with the same Pfam domain, CDS with coverage >0.8, and also supported by our transcriptome data were kept for the next analysis. Finally, 2184 high-confidence orthologs were obtained (Table S2). **(b)** The corresponding figures show the gene structure and inserts for repetitive sequences with reciprocal best gene pairs (identity > 50.0 & align > 90.0% & e-value < 1e-10) between *Arabidopsis thaliana* (AT1G04950) and other species.

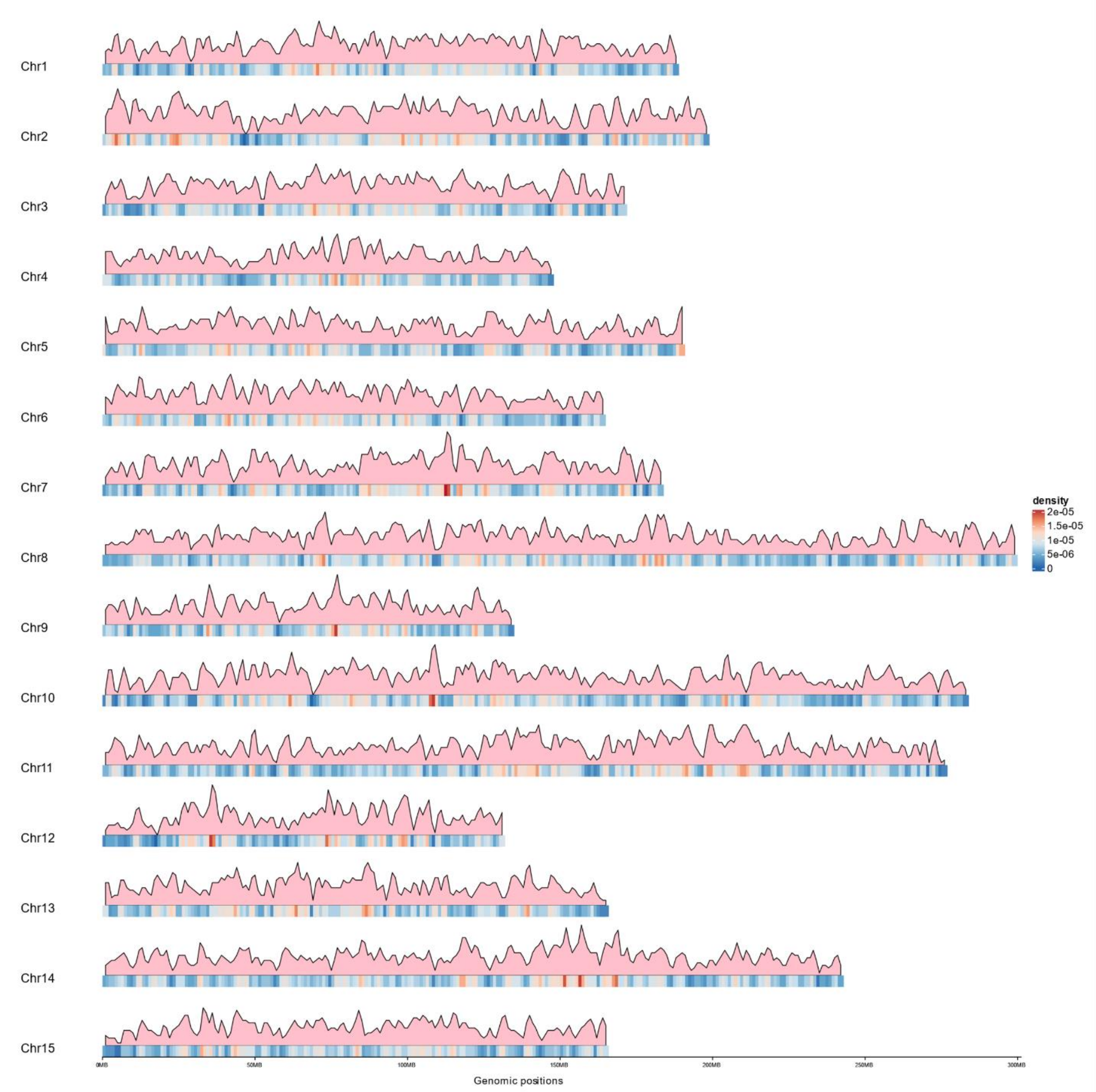

Supplementary Fig. 6. Density distribution of the full-LTR in the *C. spicatus* genome. All numbers were determined in 2-Mb windows.

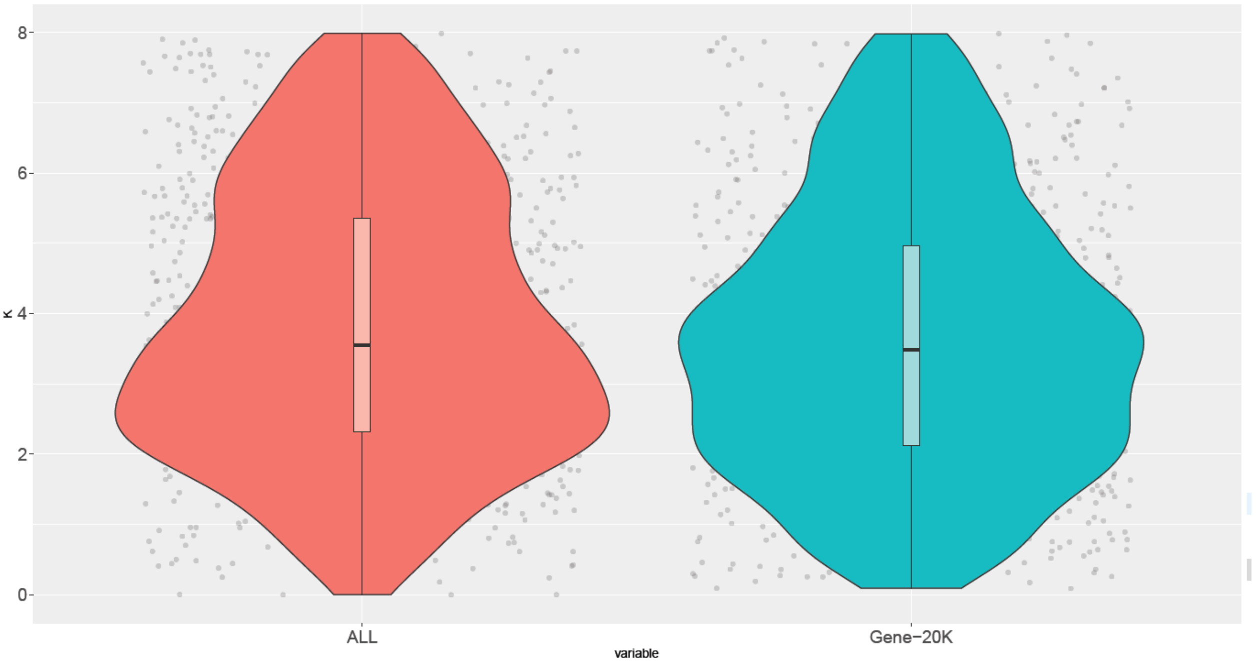

Supplementary Fig. 7. The distribution of K values of full-LTR between all genes (left) and those designated as 'long' genes (>20 K) (right) in the *C. spicatus* genome. Given the average rate of nucleotide substitution (denoted as r) and sequence distance (denoted as K), insertion date (denoted as T) can be estimated by T = K/2r. K represents the distance between two LTR pairs calculated with the Kimura two-parameter model. The full-LTR number of all and long genes were 11839 and 991, respectively, individual values are shown as dots. The median (black line, 3.55 and 3.48) is shown as a horizontal line in each violin plot. The mean's 95% CI (confidence interval) of each distribution are shown as a colored square with their corresponding error bars. The plot depicts the 25th and 75th quartile (box), the minimum and maximum (whiskers, Q1-1.5*IQR (interquartile range), Q3 + 1.5*IQR), and outliers (dots). The value of mean ± SD (standard deviation) is 3.82 ± 1.92 and 3.62 ± 1.91, respectively.

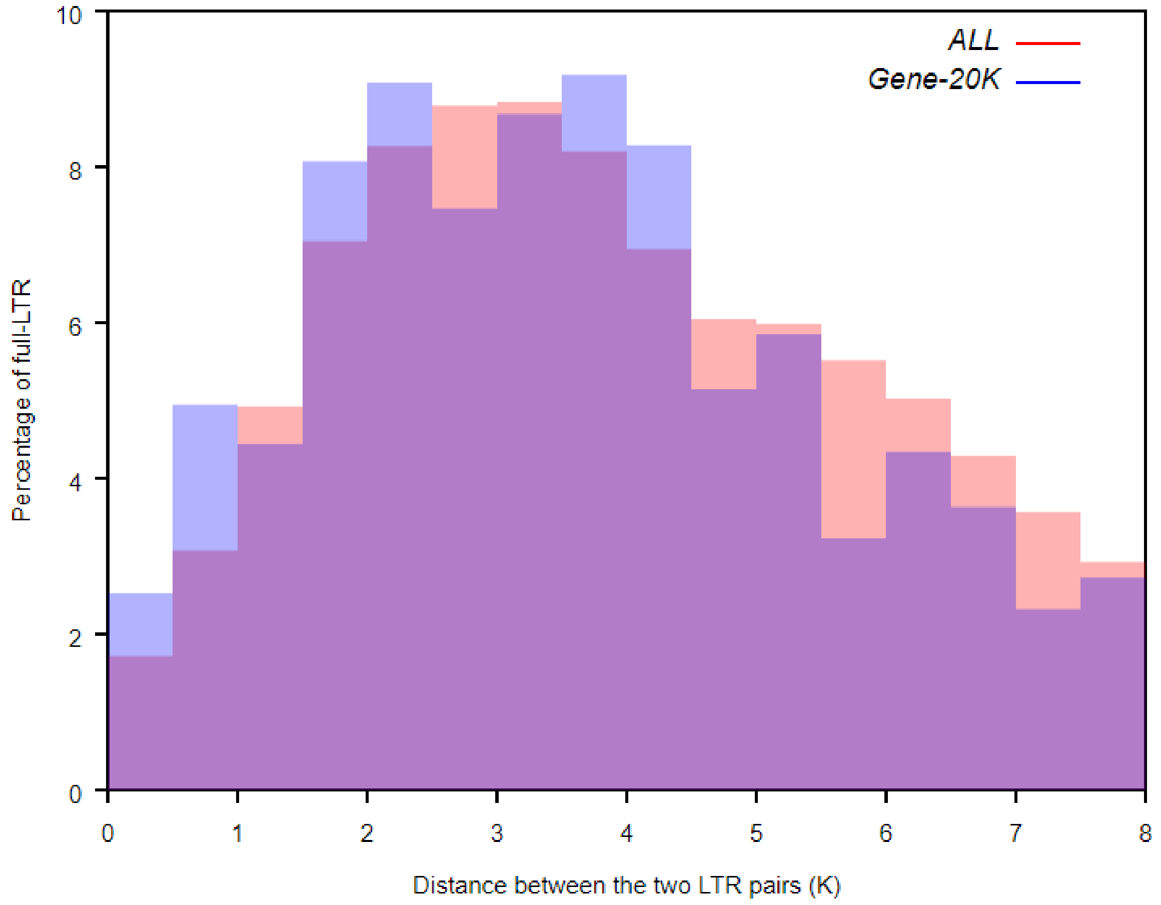

Supplementary Fig. 8. The distribution of K values of full-LTR between all and long genes (>20K) in the *C. spicatus* genome. The K represents the distance between the two LTR pairs calculated with the Kimura two-parameter model.

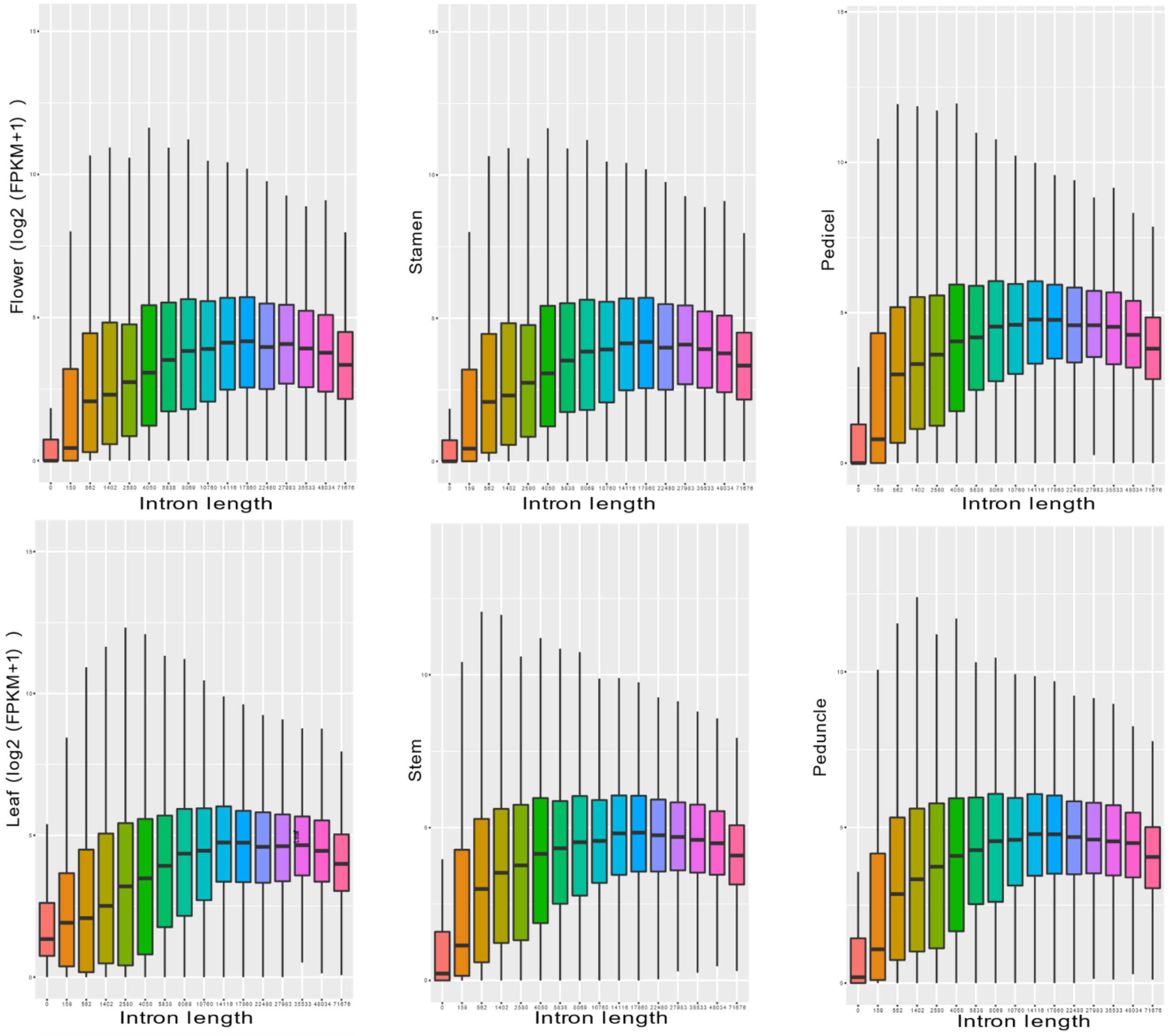

Supplementary Fig. 9. Association of intron length on expression levels in different tissues of *C. spicatus*. The x axis denotes intron length in increasing order, while the y axis displays the FPKM values. Genes with short introns more likely exhibited lower expression levels than genes with long introns. However, when the intron length was larger than 40 kb, the expression level declined. Boxplots represent median, minimum and maximum gene expression levels.

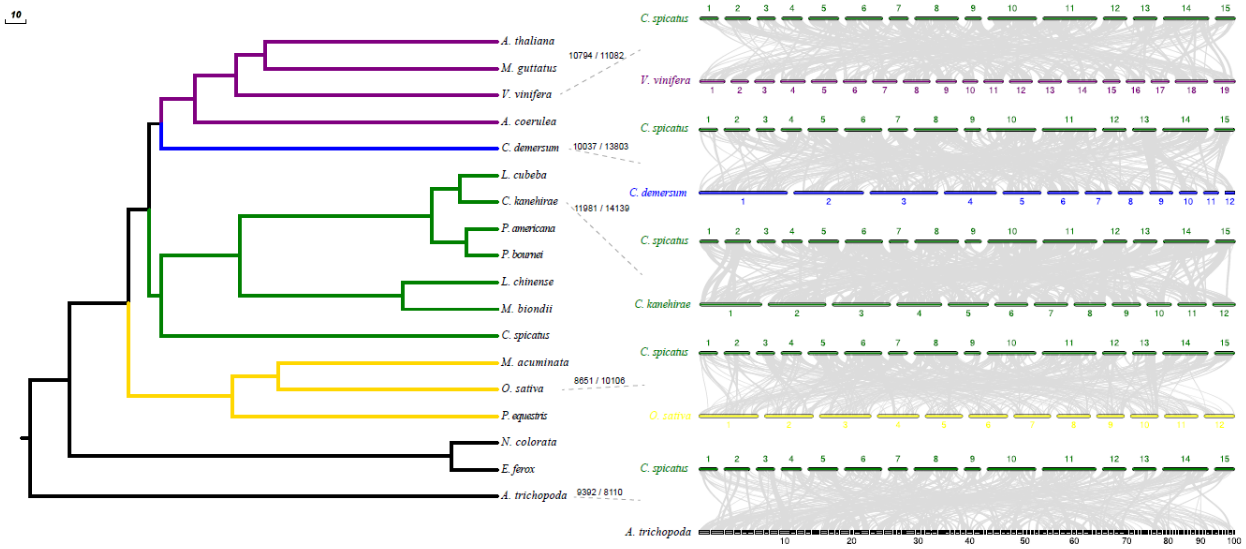

Supplementary Fig. 10. Comparison of syntenic blocks among representative genomes with *C. spicatus*. The numbers over the dashed arrow indicate genes of the two compared genomes in the syntenic blocks. The first number is for *Chloranthus*, and the second number is for the species with which it is being compared.

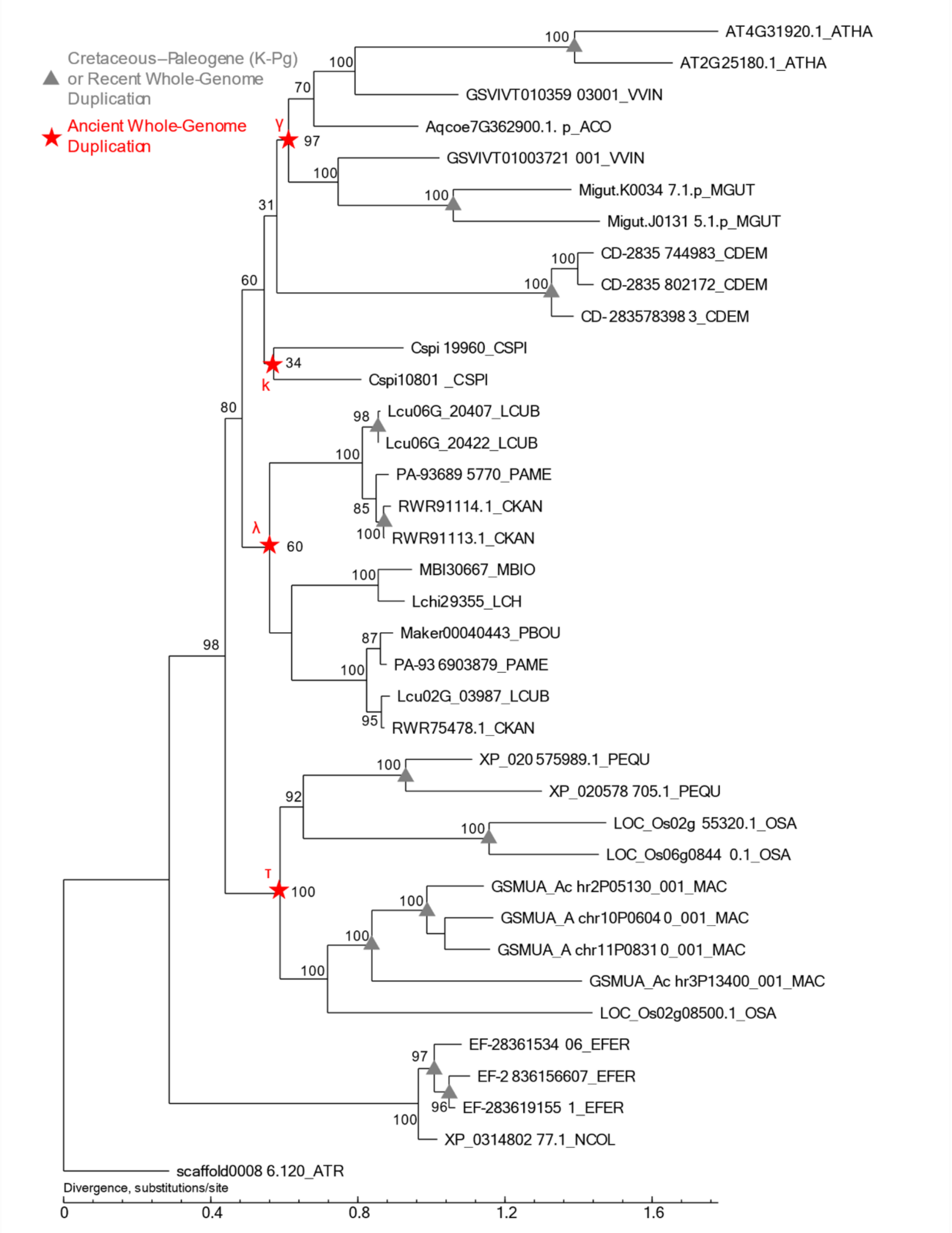

Supplementary Fig. 11. Phylogeny of the *Arabidopsis* response regulator gene family (OrthoGroup1633) showing retention of duplicate genes after ancient WGDs that occurred ~100-120 million years ago. The phylogenetic tree was constructed using IQTREE. Red stars indicate duplications retained from the ancient wave of WGDs ~120 million years ago. Gray upper triangles indicate duplications retained from the Cretaceous-Paleogene WGDs ~66 million years ago. Numbers on branches show bootstrap support values.

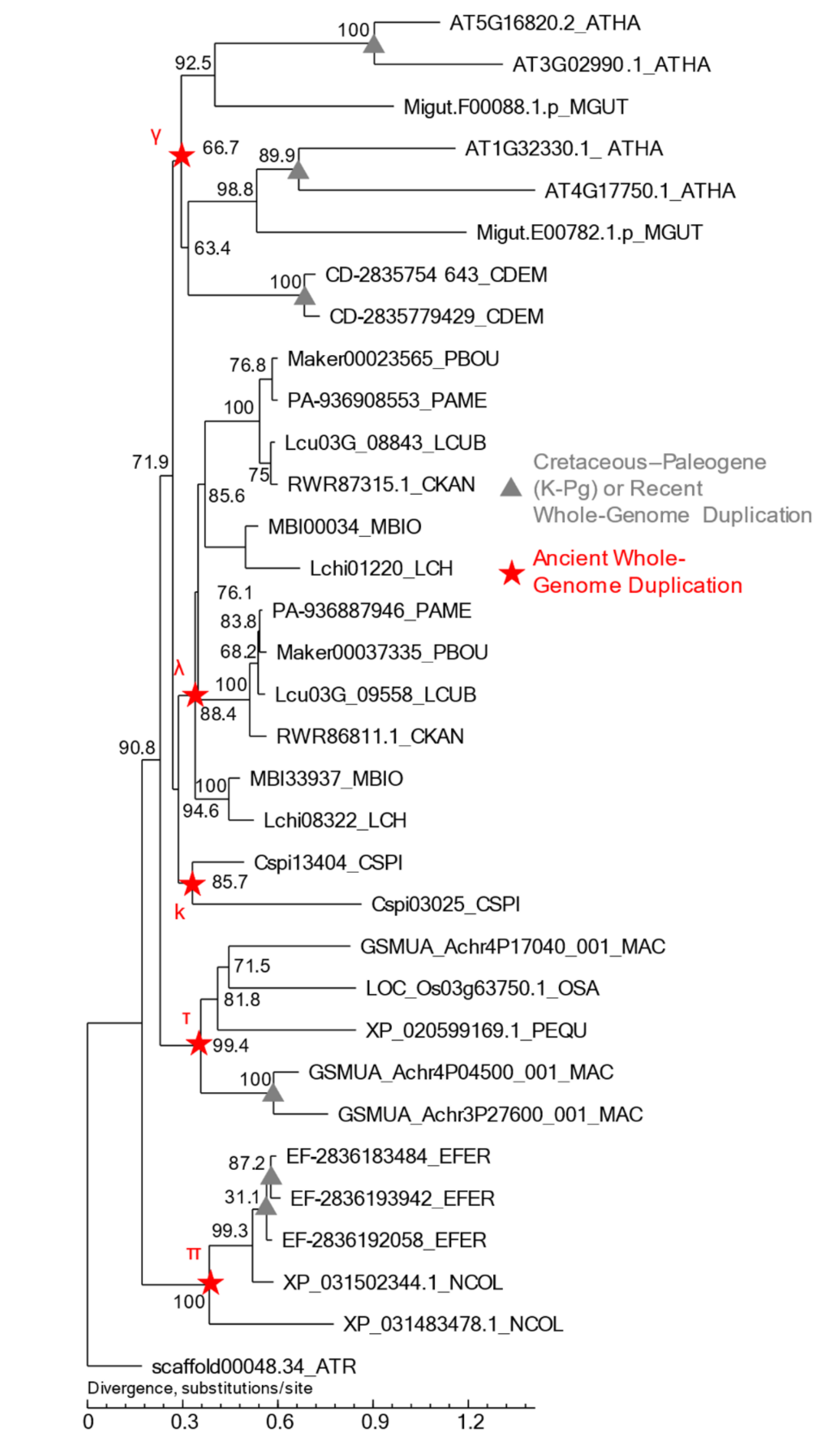

Supplementary Fig. 12. Phylogeny of the Heat Shock Transcription Factor gene family (OrthoGroup1743) showing the preferential retention of duplicate genes after ancient whole-genome duplications around 100-120 million years ago. Red stars indicate duplications retained from the ancient wave of whole-genome duplications around 120 million years ago. Gray upper triangles indicate duplications retained from the Cretaceous-Paleogene whole-genome duplications around 66 million years ago. Numbers on branches show the bootstrap supporting values.

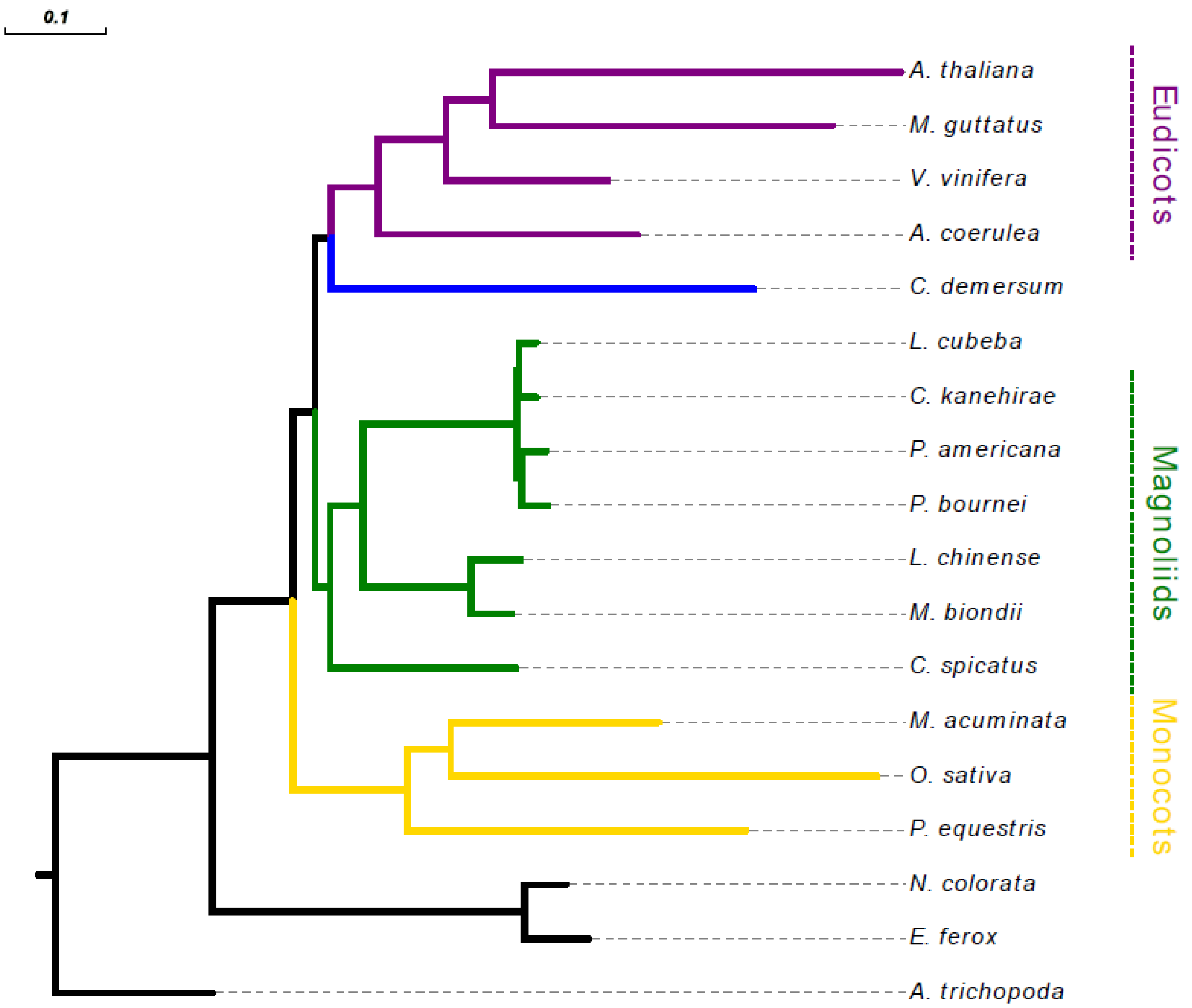

Supplementary Fig. 13. Phylogenetic trees based on analysis of the 2,329 low-copy nuclear (LCN) from 18 land plant species. Protein sequences of the LCGs were separately aligned, trimmed, and then were converted to DNA to infer single-gene phylogenies with IQ-TREE. Tree topology is supported by 100% bootstrap values.

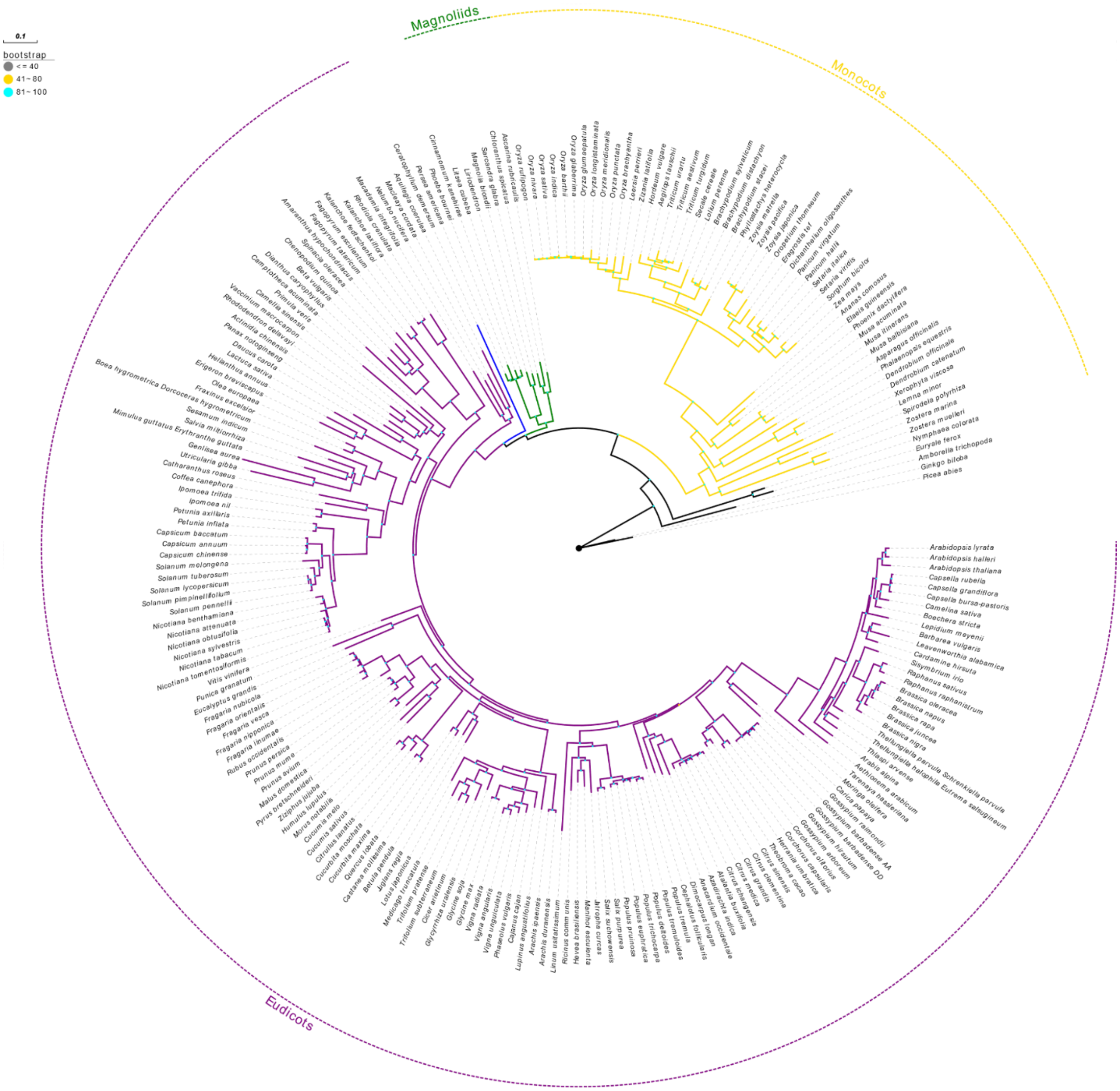

Supplementary Fig. 14. Phylogenetic tree based on 612 low-copy nuclear (LCN) in 225 land plant species. Protein sequences of the LCGs were separately aligned, trimmed and then were converted to DNA to infer single-gene phylogenies with IQ-TREE. Bootstrap values are shown in three ranges as indicated in upper left.

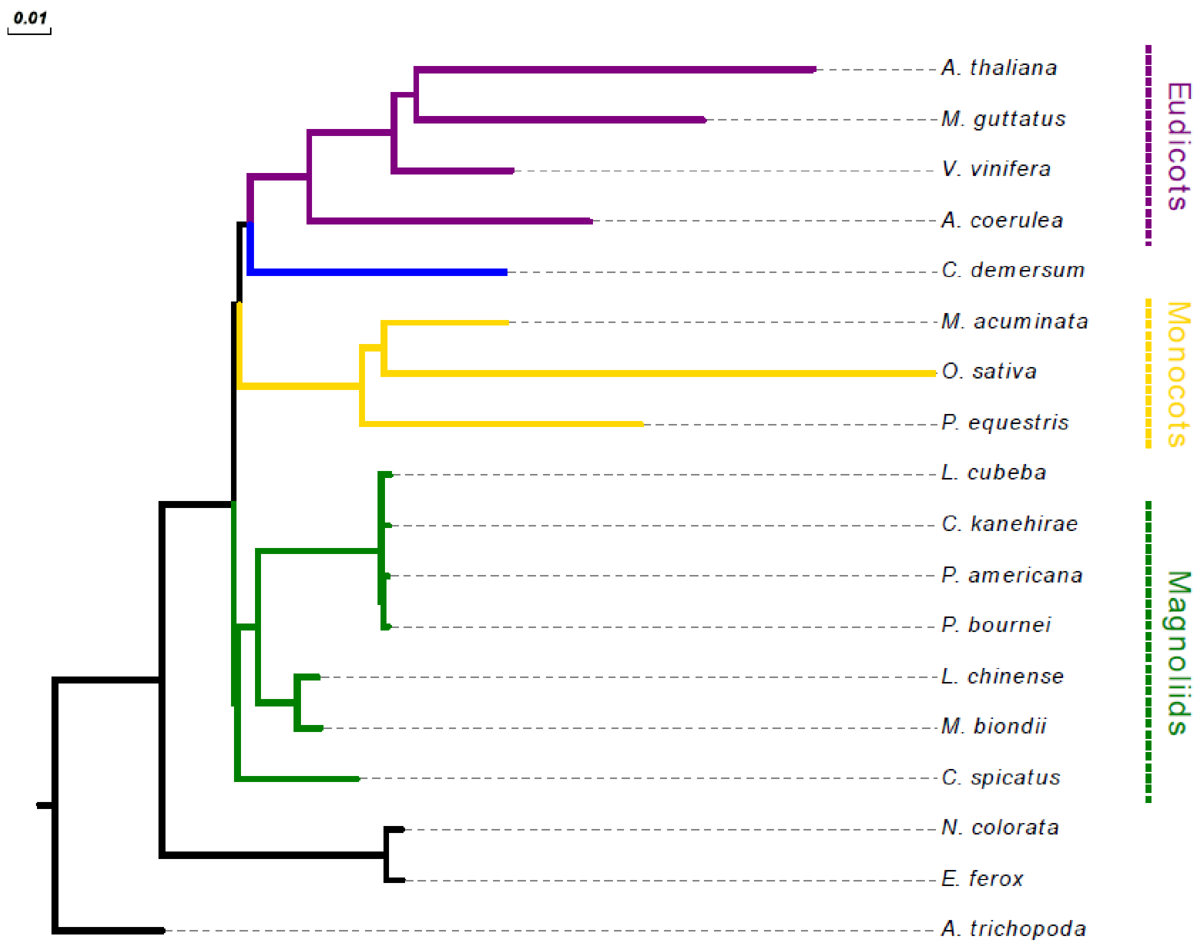

Supplementary Fig. 15. Phylogenetic trees based on 80 chloroplast genes of 18 land plant species. cpDNAs were separately aligned, trimmed and were used to infer the tree with RAxML. Tree topology is supported by 100% bootstrap values. *A. trichopoda* was used as an outgroup species.

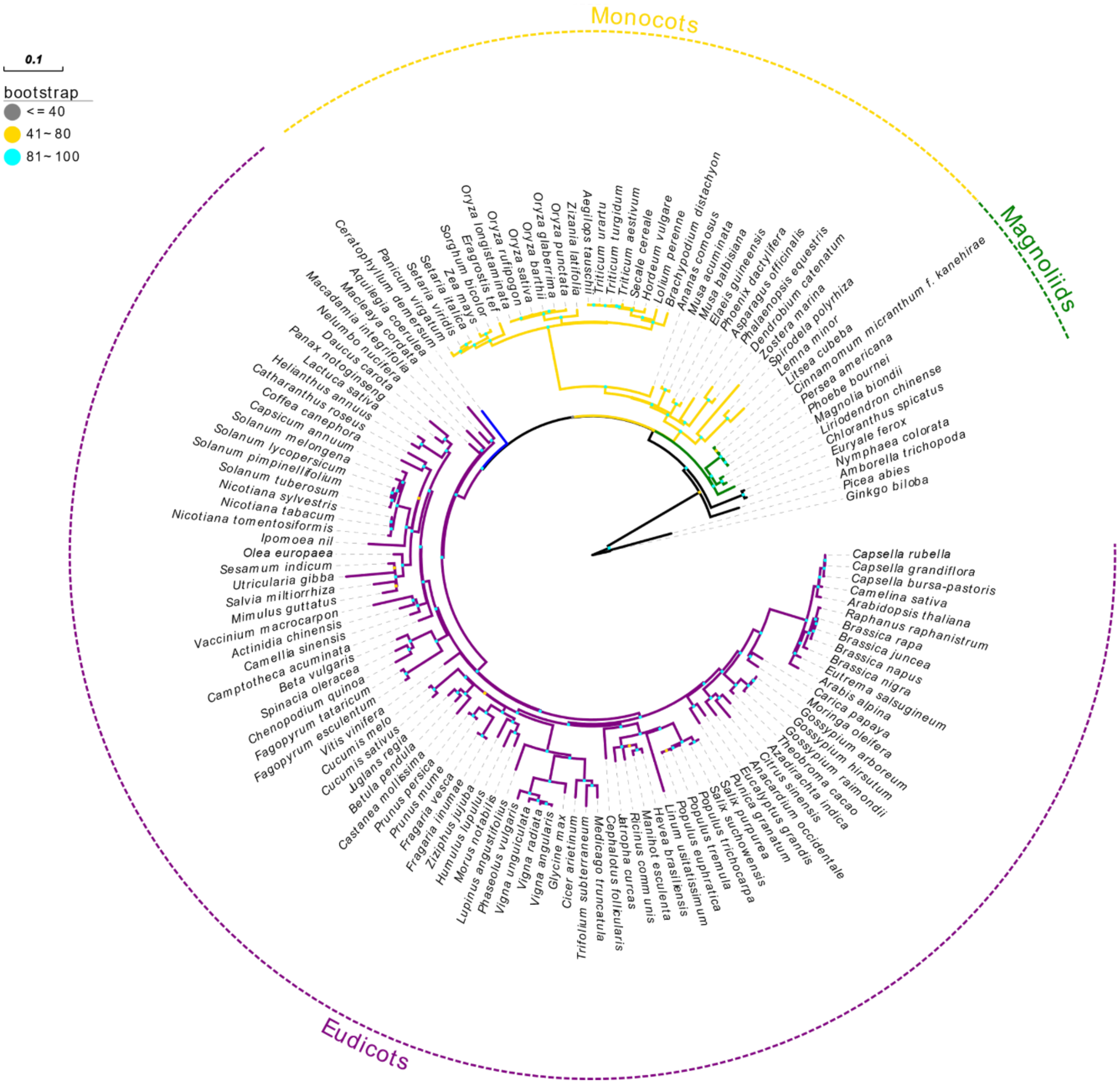

Supplementary Fig. 16. Phylogenetic tree based on analysis of 80 chloroplast genes from 134 land plant species. cpDNAs were separately aligned, trimmed and were used to infer the tree with RAxML. Tree topology is supported by 100% bootstrap values. *Ginkgo* and *Picea* were used as outgroups. Source data are provided as a Source Data file.

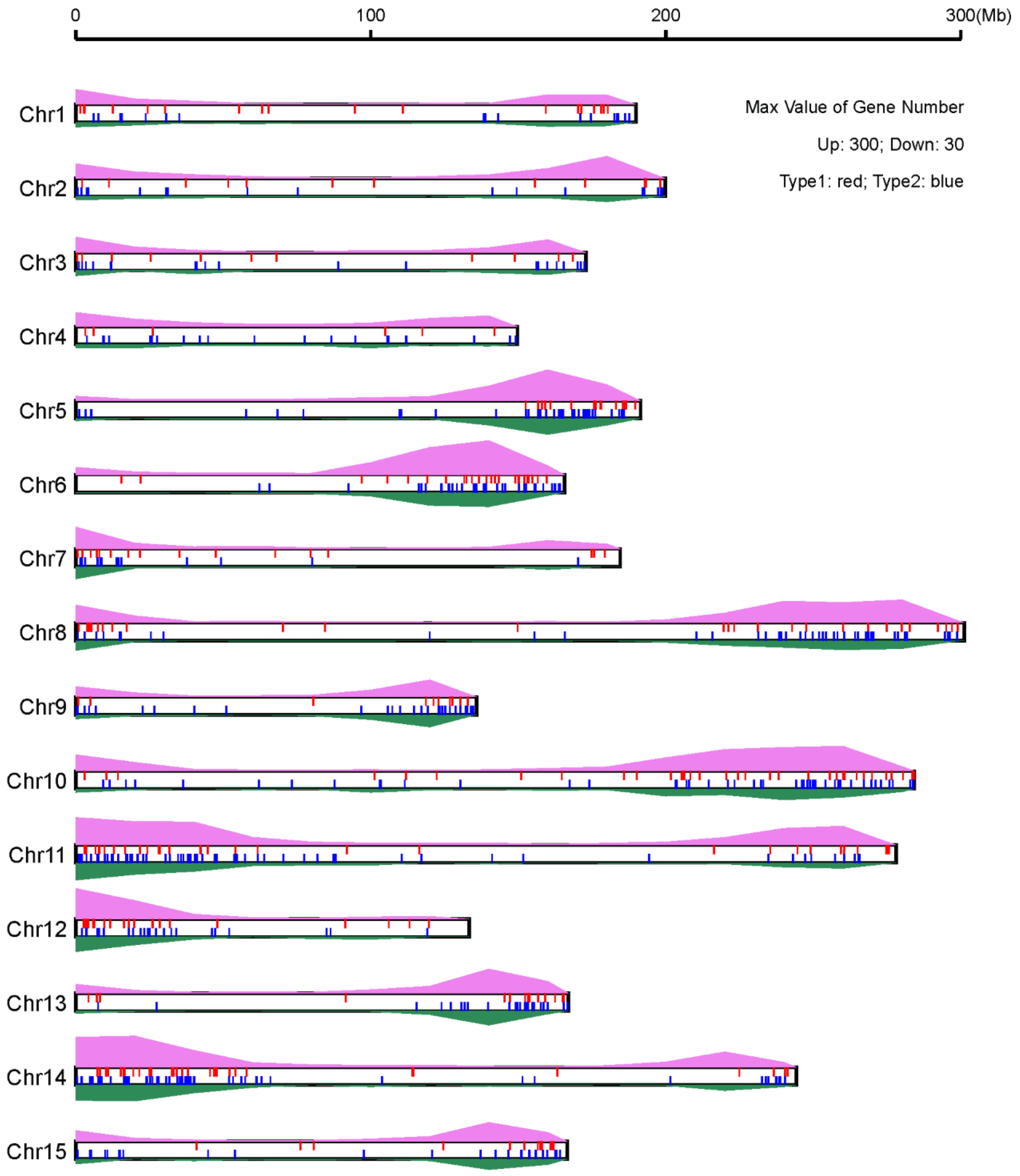

Supplementary Fig. 17. Distribution of type 1 and type 2 genes on each chromosome of *C. spicatus*. Here Type I represents the Chloranthales-magnoliids clade sister to all other Mesangiospermae, while Type II represents Chloranthales-magnoliids clade plus eudicots forming a sister group with monocots. Genes that supported both Type I (blue dot) and Type II (red dot) topologies were evenly distributed on the 15 chromosomes**.**

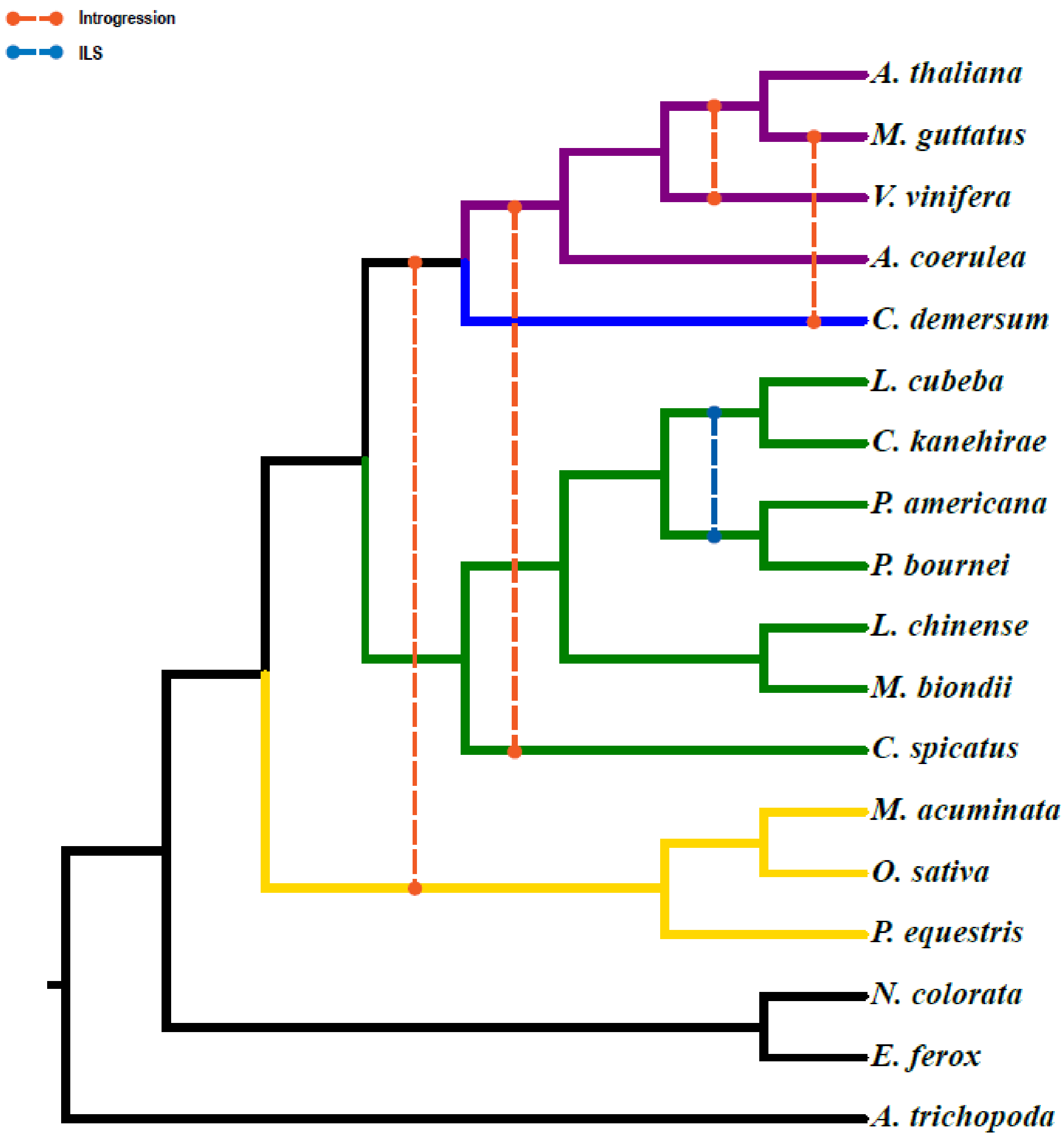

Supplementary Fig. 18. Phylogenetic incongruence in angiosperms inferred by QuIBL. Introgression (red) and ILS (blue) events are shown as broken lines on the tree. Extensive introgression events were found between monocots and eudicots or between monocots and magnoliids, and this suggested an ancient introgression event. Other introgression events were also found within eudicots, while ILS events mainly occurred within magnoliids.

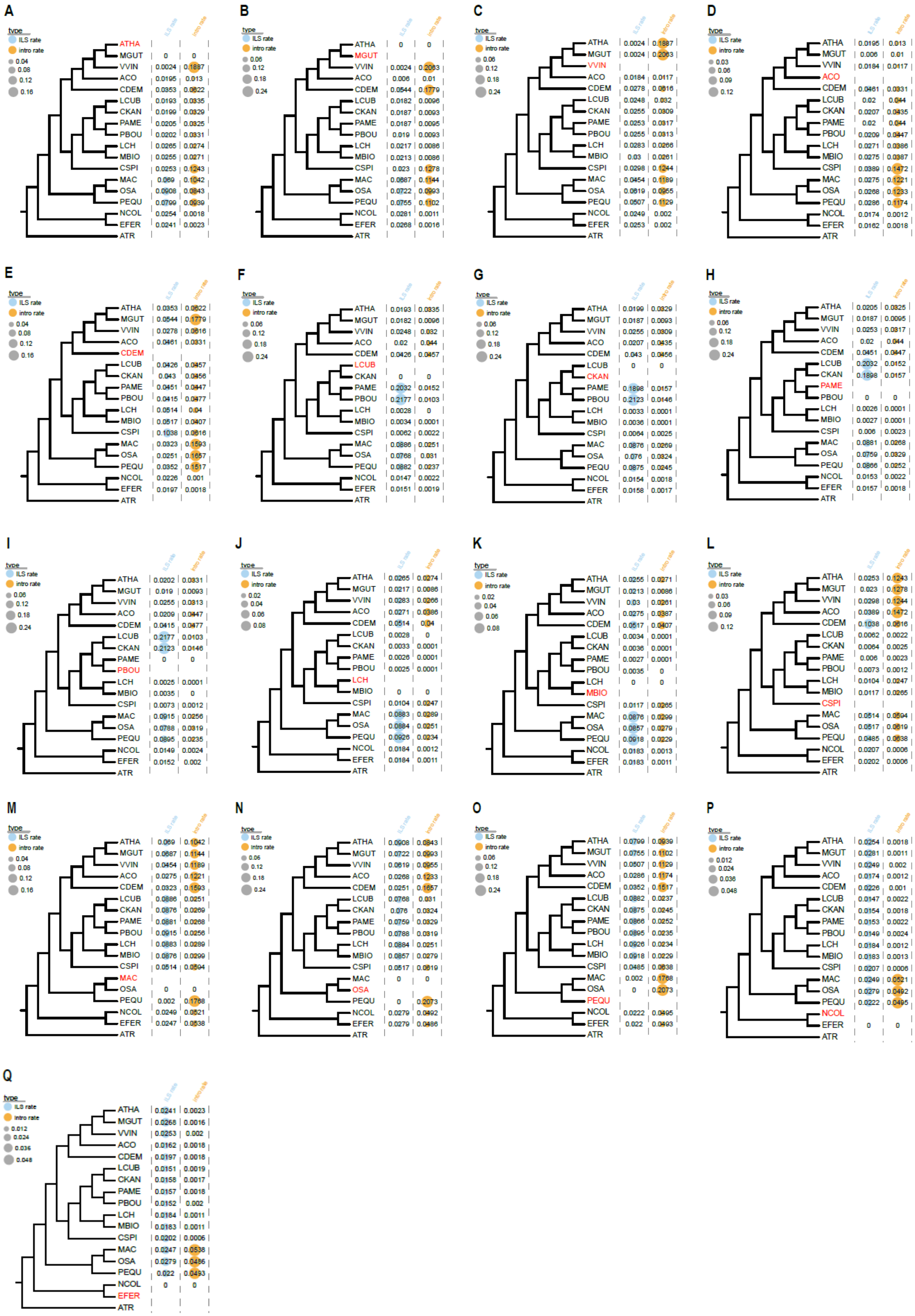
A
B
C
D
E
F
G
H
I
J
K
L
M
N
O
P
Q
type
ILS rate
intro rate
ATHA
MGUT
VVIN
ACO
CDEM
LCUB
CKAN
PAME
PBOU
LCH
MBIO
CSPI
MAC
OSA
PEQU
NCOL
EFER
ATR

Supplementary Fig. 19. Hybridization and ILS ratio were calculated between reference species and others by discordant triplets. For each subfigure (A-Q), the species marked in red was used as a reference. The numbers following each species represent the introgression and ILS ratio, respectively, between the reference and each species. The size of the blue and orange circles reflects the probability of ILS and hybridization, respectively. The conclusion corresponds to results obtained in Supplementary Fig. 13.

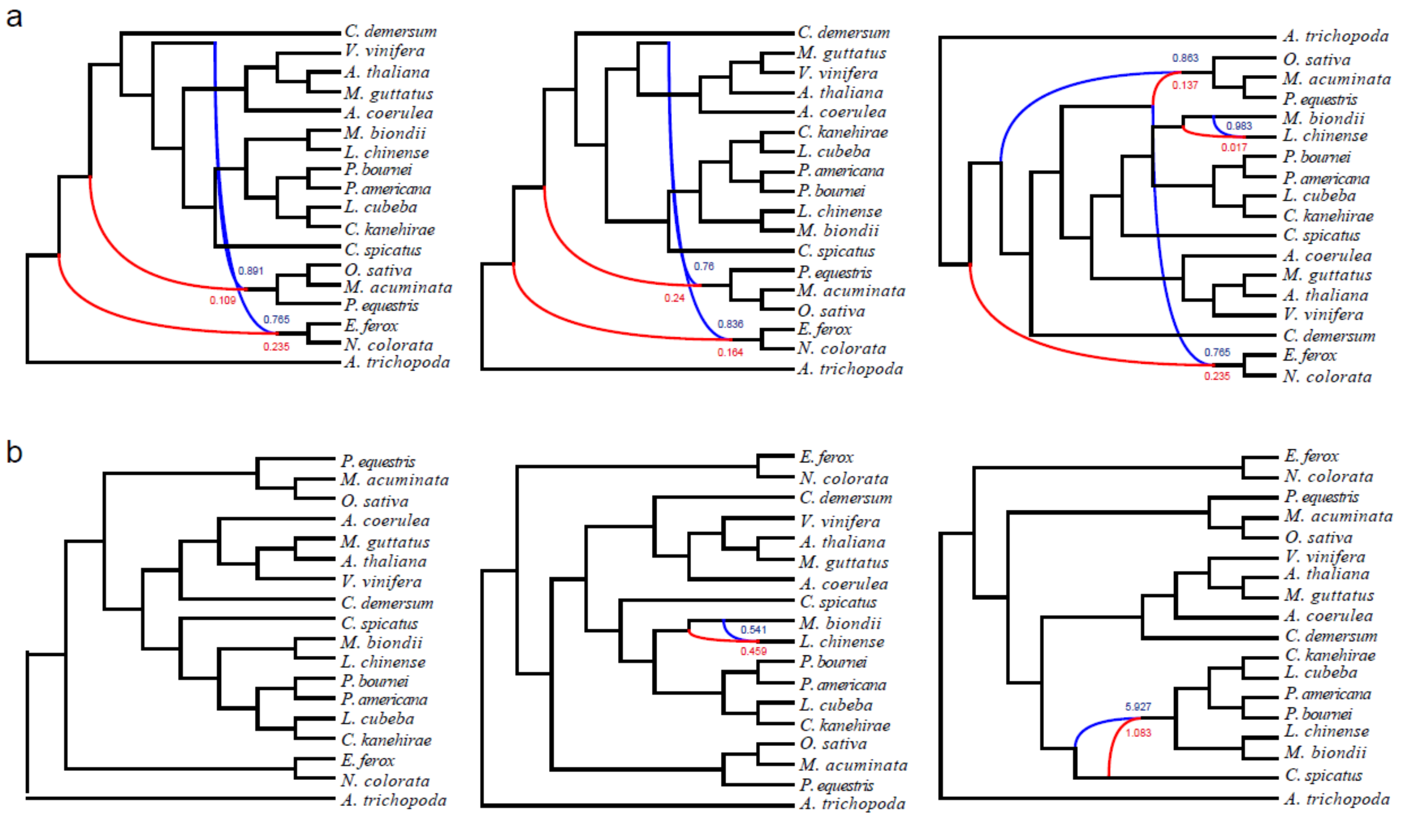

Supplementary Fig. 20. Analyzing and reconstructing reticulate evolutionary relationships with PhyloNet. (a) The best network from the five maximum reticulation events (AICs = 50.78; BICs = 30.52). The shape, or topology, of the phylogenetic network is a rooted, directed, and acyclic graph depicting the same network in terms of a backbone tree (the solid black lines) and a set of reticulation edges (the red and blue arrows). Left to right is showing the top three inferred networks (total log probability -897796.5, -898015.6, -898153.3). The inheritance probabilities are shown on the reticulation edges. **(b)** The coalescent trees were built under the species tree with ILS as the underlying source of conflict to examine potential erroneously inferring hybridization. The ASTRAL tree of 18 species was utilized as input, and 2329 trees (the same number as empirical gene trees) were simulated using DendroPy, with 10 replicates. The top three inferred networks (total log probability -1025116.4, -1025116.4, -1025128.9) are shown from left to right. The inheritance probabilities are shown on the reticulation edges (the red and blue arrows).

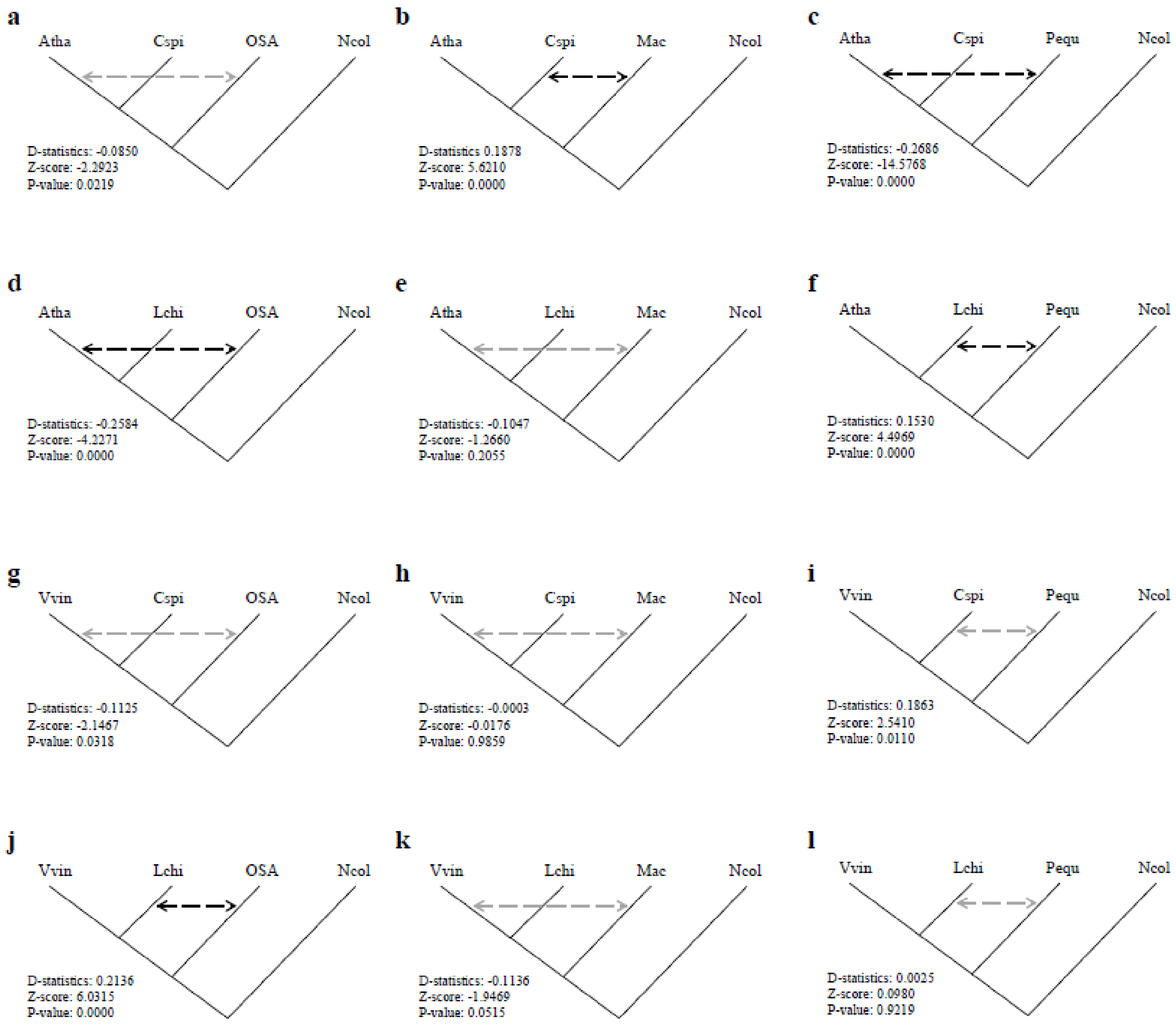

Supplementary Fig. 21. The ABBA-BABA analysis of introgression for *C. spicatus*. (a-l) The broken line in each tree represents the introgression events inferred with ABBA-BABA D-statistics. Black broken lines represent significant results (Z-score>3), and gray lines mark non-significant results in the figure (Z-score<3).

a.

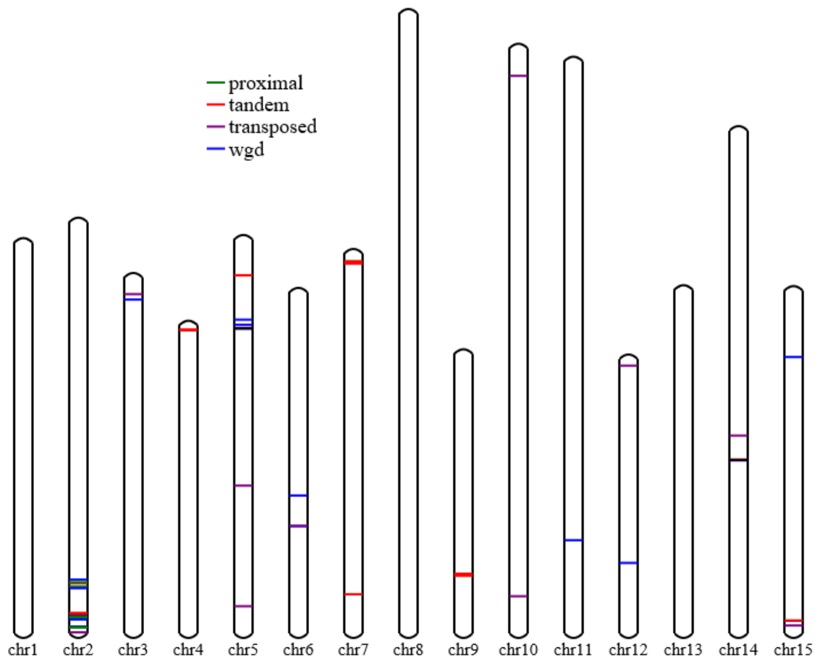

b.

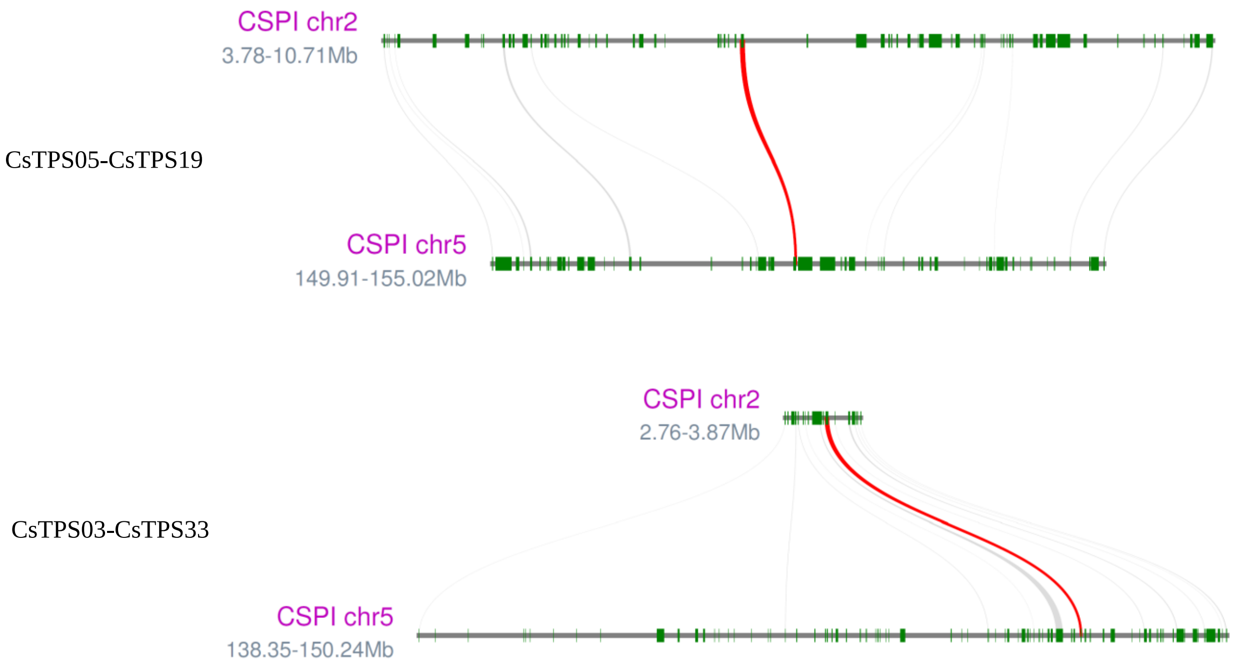

Supplementary Fig. 22. The distribution and gene replication of CsTPSs caused by an ancient WGD event

a) The distribution of CsTPSs in chromosomes; b) Gene replication produced by WGD event. Here CsTPS03 and CsTPS33, CsTPS05 and CsTPS19 are shown as representative examples.

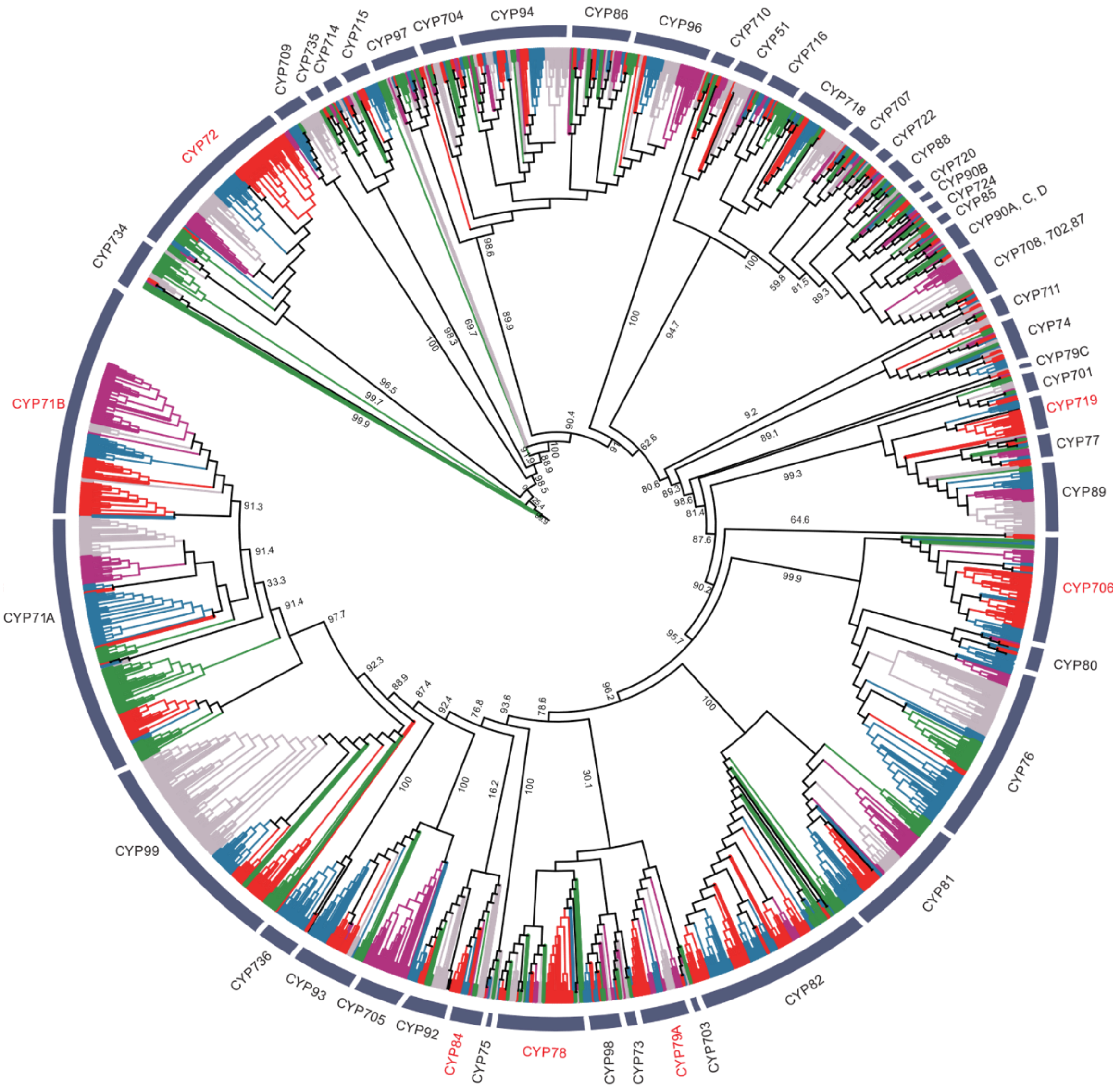

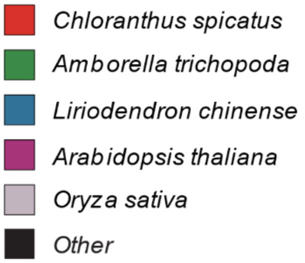

Family type	*A. trichopoda*	*L. chinense*	*C. spicatus*	*A. thaliana*	*O. sativa*
CYP71B	0	14	30	36	7
CYP84	2	4	7	2	4
CYP706	2	15	31	7	2
CYP78	7	7	20	6	9
CYP79A	0	0	9	0	0
CYP72	7	18	36	9	15
CYP719	0	4	13	0	0

Supplementary Fig. 23. The phylogenetic tree of cytochrome P450 gene family Each color represents individual species, and the table shows the gene copy number of each expanded gene families in *C. spicatus*. pfam (PF00067) was used to search the protein sequences of the five species with E-value cut off of 1e-05.

Supplementary references

[1] Chase, M. W. *et al*. An update of the Angiosperm Phylogeny Group classification for the orders and families of flowering plants: APG IV. *Botanical Journal of the Linnean Society* **181**, 1-20 (2016).

[2] Moore, M. J., Bell, C. D., Soltis, P. S. & Soltis, D. E. Using plastid genome-scale data to resolve enigmatic relationships among basal angiosperms. *P Natl Acad Sci USA* **104**, 19363-19368 (2007).

[3] Moore, M. J., Soltis, P. S., Bell, C. D., Burleigh, J. G. & Soltis, D. E. Phylogenetic analysis of 83 plastid genes further resolves the early diversification of eudicots. *P Natl Acad Sci USA* **107**, 4623-4628 (2010).

[4] Moore, M. J. *et al*. Phylogenetic analysis of the plastid inverted repeat for 244 species: Insights into deeper-level angiosperm relationships from a long, slowly evolving sequence region. *Int J Plant Sci* **172**, 541-558 (2011).

[5] Yin -Long, Q. *et al*. Angiosperm phylogeny inferred from sequences of four mitochondrial genes. *Journal of Systematics and Evolution* **48**, 391-425 (2010).

[6] Endress, P. K. & Doyle, J. A. Reconstructing the ancestral angiosperm flower and its initial specializations. *American Journal of Botany* **96**, 22-66 (2009).

[7] Zhang, N., Zeng, L., Shan, H. & Ma, H. Highly conserved low -copy nuclear genes as effective markers for phylogenetic analyses in angiosperms. *New Phytologist* **195**, 923-937 (2012).

[8] Barkman, T. J. *et al*. Independent and combined analyses of sequences from all three genomic compartments converge on the root of flowering plant phylogeny. *P Natl Acad Sci USA* **97**, 13166-13171 (2000).

[9] Qiu, Y. L. *et al*. Phylogenetic analyses of basal angiosperms based on nine plastid, itochondrial, and nuclear genes. *Int J Plant Sci* **166**, 815-842 (2005).

[10] Qiu, Y.-L. *et al*. The earliest angiosperms: evidence from mitochondrial, plastid and nuclear genomes. *Nature* **402**, 404 (1999).

[11] Zanis, M. J., Soltis, D. E., Soltis, P. S., Mathews, S. & Donoghue, M. J. The root of the angiosperms revisited. *Proc Natl Acad Sci U S A* **99**, 6848-6853 (2002).

[12] Hilu, K. W. *et al*. Angiosperm phylogeny based on matK sequence information. *American Journal of Botany* **90**, 1758-1776 (2003).

[13] Qiu, Y. L. *et al*. Reconstructing the basal angiosperm phylogeny: evaluating information content of mitochondrial genes. *Taxon* **55**, 837-856 (2006).

[14] Goremykin, V. V. *et al*. The evolutionary root of flowering plants. *Syst Biol* **62**, 50-61 (2013).

[15] Li H-T, *et al*. Origin of angiosperms and the puzzle of the Jurassic gap. *Nature Plants* **5**, 461-470 (2019).

[16] Yang L, *et al*. Phylogenomic insights into deep phylogeny of Angiosperms based on broad nuclear gene sampling. *Plant Communications* **1**, 100027 (2020).

[17] Leebens-Mack JH, *et al*. One thousand plant transcriptomes and the phylogenomics of green plants. *Nature* **574**, 679–685 (2019).

[18] Chen J, *et al*. Liriodendron genome sheds light on angiosperm phylogeny and species–pair differentiation. *Nature Plants* **5**, 18-25 (2019).
