# Supplementary material for: Chloranthus genome provides insights into the early diversification of angiosperms
Source: Nat Commun. 2021 Nov 26;12:6930. doi: 10.1038/s41467-021-26922-4 (PMC8626473; doi:10.1038/s41467-021-26922-4)
Supplement: Supplementary file 3 — Description of Additional Supplementary Files [file 41467_2021_26922_MOESM3_ESM.pdf]

Description of Additional Supplementary Files

Supplementary Data 1. Genome size estimation using 17-mer analysis of *Chloranthus spicatus*, and summary statistics of genome short-read/long read sequencing data for *Chloranthus spicatus*.

Supplementary Data 2. Comparison of genome and gene Characteristic of the *Chloranthus spicatus* genomes with other genomes.

Supplementary Data 3. Statistics of the de novo genome assembly of *Chloranthus spicatus*.

Supplementary Data 4. Evaluation of completeness of gene set for *Chloranthus spicatus* using the BUSCO database.

Supplementary Data 5. Overlap between tandem repeats and protein coding genes in selected species, and Repeat elements annotated in the *Chloranthus spicatus* genome.

Supplementary Data 6. Repeat elements annotated in the *Chloranthus spicatus* genome.

Supplementary Data 7. Data on all 18 species used for the evolutionary analysis and the list of 218 species used for nuclear phylogenetic analyses in this study.

Supplementary Data 8. Comparison of high-ortholog gene (HOG) Characteristic of the *Chloranthus spicatus* genomes with other genomes.

Supplementary Data 9. Comparison of high-ortholog gene (HOG) size of the *Chloranthus spicatus* genomes with other genomes.

Supplementary Data 10. Statistics of Syntenic Blocks between different representative genome with scaffold and chromosome level.

Supplementary Data 11. Ks distribution related to duplication events within representative genome and between genomes.

Supplementary Data 12. Random distribution with chi-square test for Type1- and Type 2-Tree genes.

Supplementary Data 13. Random distribution with chi-square test for Type1/2-Tree genes.

Supplementary Data 14. Model selection of PhyloNet analysis.

Supplementary Data 15. TPS genes in *Chloranthus spicatus*.

Supplementary Data 16. Numbers of TPS subfamilies in 18 species.

Supplementary Data 17. The GO enrichment analysis of ancestral gene ($P<0.05$).

Supplementary Data 18. TPSs duplication types.

Supplementary Data 19. The statistic of genes involved in MEP and MVA pathway.

Supplementary Data 20. Related enzymes of MEP and MVA pathway.

Supplementary Data 21. Resistance gene (Rgenes) present in *Chloranthus spicatus* and other genomes.

Supplementary Data 22. Functional enrichment of unique and tandem genes of *Chloranthus spicatus* genome.
