# Supplementary material for: Chloranthus genome provides insights into the early diversification of angiosperms
Source: Nat Commun. 2021 Nov 26;12:6930. doi: 10.1038/s41467-021-26922-4 (PMC8626473; doi:10.1038/s41467-021-26922-4)
Supplement: Supplementary file 26 — Reporting Summary [file 41467_2021_26922_MOESM26_ESM.pdf]

nature portfolio

Corresponding author(s): Huan Liu
Douglas E. Soltis

Last updated by author(s): Sep 15, 2021

Reporting Summary

Nature Portfolio wishes to improve the reproducibility of the work that we publish. This form provides structure for consistency and transparency in reporting. For further information on Nature Portfolio policies, see our Editorial Policies and the Editorial Policy Checklist.

Statistics

For all statistical analyses, confirm that the following items are present in the figure legend, table legend, main text, or Methods section.

n/a	Confirmed	
☒	☐	The exact sample size (*n*) for each experimental group/condition, given as a discrete number and unit of measurement
☒	☐	A statement on whether measurements were taken from distinct samples or whether the same sample was measured repeatedly
☐	☒	The statistical test(s) used AND whether they are one- or two-sided<br>*Only common tests should be described solely by name; describe more complex techniques in the Methods section.*
☒	☐	A description of all covariates tested
☒	☐	A description of any assumptions or corrections, such as tests of normality and adjustment for multiple comparisons
☒	☐	A full description of the statistical parameters including central tendency (e.g. means) or other basic estimates (e.g. regression coefficient) AND variation (e.g. standard deviation) or associated estimates of uncertainty (e.g. confidence intervals)
☐	☒	For null hypothesis testing, the test statistic (e.g. *F*, *t*, *r*) with confidence intervals, effect sizes, degrees of freedom and *P* value noted<br>*Give P values as exact values whenever suitable.*
☐	☒	For Bayesian analysis, information on the choice of priors and Markov chain Monte Carlo settings
☒	☐	For hierarchical and complex designs, identification of the appropriate level for tests and full reporting of outcomes
☒	☐	Estimates of effect sizes (e.g. Cohen's *d*, Pearson's *r*), indicating how they were calculated

Our web collection on statistics for biologists contains articles on many of the points above.

Software and code

Policy information about availability of computer code

Data collection	No software was used for data collection
Data analysis	The list of Software used in this study are as follows:<br>Trimmomatic (v0.40),<br>MinKNOW package (https://github.com/nanoporetech/minknow_api),<br>GenomeScope,<br>NextDenovo assembler (v2.2),<br>NextPolish (v1.3.0),<br>purge_dups (v1.2.3),<br>BLAST (v2.2.29),<br>BWA (v.2.21),<br>BLAT (v.36),<br>Trinity (v2.6.6),<br>Juicer V3,<br>3ddna (v180922),<br>Juicebox assembly tools (v1.11.08),<br>Tandem Repeats Finder (4.07),<br>RepeatMasker (4.0.5),<br>RepeatProteinMask,<br>LTR_retriever,<br>LTR_FINDER (1.0.6),<br>RepeatModeler2,

MITE-hunter,
DANTE-Protein Domain Finder,
RepeatExplorer server,
Augustus (3.0.3),
GlimmerHMM (3.0.1),
SNAP (version 11/29/2013),
TBLASTN (2.2.18),
GeneWise (2.2.0),
Hisat2 (v2.0.4),
StringTie (v1.2.2),
PASA (version 2.0.2),
MAKER pipeline (v2),
Tophat2,
Samtools (v.0.1.19),
InterProScan (v5.28-67.0),
iTAK (v1.6),
OrthoMCL (1.4),
Café (v4),
MAFFT (v.7.310),
PAL2NAL (v14),
trimal (v1.4.1),
IQTREE (v1.6.12),
ModelFinder,
Pheatmap package (v1.0.12),
ASTRAL (v.5.6.1),
OrthoFinder (v2.2.7),
RAxML (v. 8.2.12),
InterProScan (v5.28-67.0),
iTAK (v1.6),
OrthoMCL (1.4),
Café (v4),
MAFFT (v.7.310),
PAL2NAL (v14),
trimal (v1.4.1),
IQTREE (v 1.6.12),
ModelFinder,
ASTRAL (v.5.6.1),
OrthoFinder (v2.2.7),
RAxML (v. 8.2.12),
BUSCO (v3.0.2),
MCMCTREE (v4.5),
PAML ,
MUSCLE (v3.8.31),
MCScanX,
QuIBL,
hmmer(v3.2.1)

For manuscripts utilizing custom algorithms or software that are central to the research but not yet described in published literature, software must be made available to editors and reviewers. We strongly encourage code deposition in a community repository (e.g. GitHub). See the Nature Portfolio guidelines for submitting code & software for further information.

Data

Policy information about availability of data

All manuscripts must include a data availability statement. This statement should provide the following information, where applicable:

- Accession codes, unique identifiers, or web links for publicly available datasets
- A description of any restrictions on data availability
- For clinical datasets or third party data, please ensure that the statement adheres to our policy

The data supporting the findings of this work are available within the paper and its Supplementary Information files. A reporting summary for this article is available as a Supplementary Information file. The genome and transcriptome data generated in this study have been deposited in the NCBI Sequence Read Archive database under accession code PRJNA770110 [https://www.ncbi.nlm.nih.gov/bioproject/?term=PRJNA770110], and CNSA of CNGBdb with accession code CNP0001771 [https://db.cngb.org/search/?q=CNP0001771].

Field-specific reporting

Life sciences study design

All studies must disclose on these points even when the disclosure is negative.

Sample size	One sample's genome was sequenced. The young leaf, stem, and pedicel tissues of C. spicatus were used for transcriptome sequencing in 2-4 biological replicates. For phylogenetic tree construction different numbers of species were selected as described in method section "Phylogenetic analyses"
Data exclusions	1. For the genome assembly, we searched against bacterial database to rule out any potential bacterial contamination. Contigs with an identity greater than 90% and an alignment of at least 80% were excluded<br><br>2. Before performing the transcriptome assembly, we retrieved high-quality reads by eliminating adaptor sequences and filtering low-quality reads using TRIMMOMATIC (v0.40).<br><br>3. To estimate the timing of whole-genome duplication events, low-copy families were exclusded based on pairwise comparison of paralog sequences.
Replication	Replication was used for the transcriptome data analysis. For phylogenetic analysis various bootstrap replicates were used as well
Randomization	As we sequenced only one genome. Data randomization was not required.
Blinding	No experimental validation works were carried out. Hence, blinding was not applicable.

Reporting for specific materials, systems and methods

We require information from authors about some types of materials, experimental systems and methods used in many studies. Here, indicate whether each material, system or method listed is relevant to your study. If you are not sure if a list item applies to your research, read the appropriate section before selecting a response.

Materials & experimental systems

n/a	Involved in the study
☒	☐ Antibodies
☒	☐ Eukaryotic cell lines
☒	☐ Palaeontology and archaeology
☒	☐ Animals and other organisms
☒	☐ Human research participants
☒	☐ Clinical data
☒	☐ Dual use research of concern

Methods

n/a	Involved in the study
☒	☐ ChIP-seq
☒	☐ Flow cytometry
☒	☐ MRI-based neuroimaging
